# Genetic evidence suggests a causal relationship linking thyroid function to osteomyelitis

**DOI:** 10.1016/j.clinsp.2026.100940

**Published:** 2026-04-23

**Authors:** Junming Huang, Mingchao Lin, Zhipeng Wang, Zhiyuan Zou, Shanhu Huang, Song Zhou

**Affiliations:** a The Orthopedic Hospital, The First Affiliated Hospital, Jiangxi Medical College, Nanchang University, Nanchang, China; b Department of Sports Medicine, Orthopedic Hospital, The First Affiliated Hospital, Jiangxi Medical College, Nanchang University, Nanchang, China; c The Key Laboratory of Spine and Spinal Cord Diseases of Jiangxi Province, Nanchang, , China; d Postdoctoral Research Station, The First Affiliated Hospital, Jiangxi Medical College, Nanchang University, Nanchang, China

**Keywords:** Mendelian randomization, Osteomyelitis, Hypothyroidism, Hyperthyroidism, Causal relationship

## Abstract

**Objective:**

The causal relationship between thyroid dysfunction and osteomyelitis was unclear. The authors used two-sample Mendelian Randomization (MR) methods to investigate the potential causal effects of hyperthyroidism and hypothyroidism on the risk of osteomyelitis.

**Method:**

The authors obtained summary-level data from large-scale genome-wide association studies for hyperthyroidism, hypothyroidism, and osteomyelitis for individuals of European ancestry. The causal analysis between thyroid dysfunction and osteomyelitis was carried out using Univariable MR (UVMR). Subsequently, Multivariable MR (MVMR) was performed to adjust for potential confounding from years of schooling, smoking initiation, and body mass index. A range of sensitivity analyses, including reverse MR, was conducted to test the robustness of the results.

**Results:**

In univariable analysis, genetically predicted hyperthyroidism (Odds Ratio [OR = 1.21], 95% Confidence Interval [95% CI 1.071‒1.374], p = 0.002) and hypothyroidism (OR = 1.140, 95% CI 1.035‒1.255, p = 0.0078) were both associated with an increased risk of osteomyelitis. After adjusting for confounders in the MVMR analysis, the association for hyperthyroidism was attenuated to non-significance (OR = 1.07, 95% CI 0.95‒1.21, p = 0.28). In contrast, the causal effect of hypothyroidism on osteomyelitis remained robust (OR = 1.15, 95% CI 1.03‒1.29, p = 0.02). Reverse MR analyses showed no evidence that osteomyelitis was a cause of either hyperthyroidism or hypothyroidism.

**Conclusion:**

This study provided genetic evidence supporting a causal effect of hypothyroidism on an increased risk of osteomyelitis. This finding provides evidence-based recommendations for closely monitoring patients with hypothyroidism to prevent the onset of osteomyelitis.

## Highlights

• Two-sample MR confirms hypothyroidism causally elevates osteomyelitis risk.
• Hyperthyroidism-osteomyelitis link is confounded by lifestyle and BMI factors.
• No reverse causality from osteomyelitis to thyroid dysfunction detected.
• Hypothyroidism patients need closer infection monitoring in orthopedic care.

## Introduction

Osteomyelitis is a pathological state distinguished by an inflammatory response in the bone tissue, which arises from the contiguous dissemination of infection from adjacent soft tissues and joints, hematogenous seeding, or direct bacterial inoculation into the bone due to trauma or surgical procedures. This condition manifests as the destruction of various skeletal structures, including trabeculae, cortical bone, bone marrow, and periosteum, resulting in localized pain, swelling, and potentially the development of sinus tracts in the skin. As one of the oldest reported diseases known to the scientific community, the incidence of osteomyelitis exhibited a continuing upward trend since the introduction of implants in orthopedic practices and the diagnosis of osteomyelitis has improved over the previous decades. In the United States, the estimated occurrence of osteomyelitis increased from 11.4 cases per 100,000 person-years during the period of 1969‒1979 to 24.4 cases per 100,000 person-years between 2000 and 2009.^1^ The conventional approach to managing osteomyelitis typically involves the use of antibiotics administered either systemically or locally, in conjunction with surgical intervention to address necrotic bone. However, the unfavorable prognosis associated with osteomyelitis, characterized by frequent relapses and progression to chronic osteomyelitis, presents considerable obstacles for both patients and surgeons.

The onset of osteomyelitis is closely tied to external infectious factors. There is a difference in the bacterial profile of the infection depending on the age of the susceptible individual, including infants, children, and adults. In general, hematogenous osteomyelitis is a predominantly pediatric disease affecting the vertebral body and epiphysis due to hematogenous spread of a single organism. For adults, traumatic osteomyelitis is the prevailing clinical archetype, which primarily arises from the localized transmission of persistent infectious agents, often following open fractures or arthroplasty procedures. In addition to pathogenic micro-organisms, the host-related factors play a substantial role in osteomyelitis evolution. Previous study has shown that osteomyelitis is more common among patients suffering from diabetes, aging, hepatitis, and immune suppression.^2^

A well-balanced immune response is essential for effectively combating infections while minimizing harm to the host. In the context of osteomyelitis, dysregulation of the immune system can significantly contribute to the disease's development and progression. A study has shown that osteomyelitis was associated with increased anti-inflammatory response and immune exhaustion.^3^ The endocrine and immune systems are linked through bidirectional communication, referred to as endocrine-immune interactions. These interactions are critical for maintaining homeostasis and responding to external and internal challenges. A recent study discussed the psycho-neuro-endocrine immune interactions in rheumatic disease, emphasizing the interplay between these systems in disease prevention and treatment.^4^ The thyroid gland, a key component of the endocrine system, produces and secretes thyroid hormones, which are iodinated derivatives of tyrosine, and play a crucial role in the regulation of various physiological processes in vivo, including growth, development, differentiation, and metabolism.^5^ Optimal thyroid function and adequate levels of thyroid hormones are imperative for the appropriate development and functioning of organs, particularly within the immune system.^6^ Emerging studies indicate that hyperthyroidism and hypothyroidism have the potential to impact various immune functions, including but not limited to antibody production, lymphocyte proliferation, phagocytosis, cytokine secretion, chemotaxis, and reactive oxygen species generation.^7^^,^^8^ Previous studies have also demonstrated a strong association between thyroid dysfunction and susceptibility to infectious diseases, including pulmonary and urinary tract infections.^8^^,^^9^ However, no studies of thyroid dysfunction and osteomyelitis have been reported to date. Thus, the authors cannot make a conclusion about the causal relationship between thyroid dysfunction and osteomyelitis.

Mendelian Randomization (MR) is a statistical technique that leverages genetic variants identified through Genome-Wide Association Studies (GWAS) as instrumental variables to ascertain causal relationships between exposure and outcome. Within MR analysis, the genetic variations (typically Single-Nucleotide Polymorphisms, SNPs) associated with the exposure of interest are randomly assigned inherited mirroring the random allocation in Randomized Controlled Trials (RCTs) at conception, and alleles are not influenced by disease, thereby mitigating potential confounding variables and preventing reverse causality in observational studies and addressing the limitations in terms of time and cost of RCTs,^10^^,^^11^ thus MR has become a valuable tool for causal inference in epidemiology.

In this study, the authors aimed to understand the potential causal link between thyroid dysfunction and osteomyelitis by conducting a two-sample MR analysis to explore the potential causal connection. The authors hypothesized that thyroid dysfunction causally increases the risk of osteomyelitis. Additionally, we examined the potential reverse causality, hypothesizing that osteomyelitis causally influences thyroid dysfunction. The primary goal was to provide robust evidence and deeper insights into the pathophysiology of osteomyelitis, laying a scientific foundation for future prevention and treatment strategies.

## Materials and methods

### Study design

The data of this manuscript were obtained from several public databases. First, the authors investigated the two-way causal link between hyperthyroidism, hypothyroidism, and osteomyelitis using a bidirectional two-sample MR (2-SMR) analysis. Second, the authors utilized Multivariate MR (MVMR) analysis to investigate the causal relationship between them after adjusting for years of schooling, smoking initiation, and Body Mass Index (BMI). This observational genetic study was designed and reported in accordance with the STROBE statement for strengthening the reporting of observational studies in epidemiology.

### GWAS data for hyperthyroidism, hypothyroidism, years of schooling, smoking initiation, BMI and osteomyelitis

This study used completely independent GWAS data for exposure and outcome phenotypes to avoid bias due to sample overlap. The GWAS data for hyperthyroidism (ebi-a-GCST90018860), hypothyroidism (ebi-a-GCST90018862), Years of schooling (ieu-a-1239), smoking initiation (ieu-b-4877), and BMI (ukb-a-248) were retrieved from the IEU database (https://gwas.mrcieu.ac.uk/). There were hyperthyroidism data for 460,499 individuals and 24,189,279 Single-Nucleotide Polymorphisms (SNPs). There were hypothyroidism data for 410,141 individuals and 24,138,872 Single-Nucleotide Polymorphisms (SNPs). There were Years of schooling data for 766,345 individuals and 10,101,242 SNPs. There were smoking initiation data for 607,291 individuals and 11,802,365 SNPs. There were BMI data for 336,107 individuals and 10,894,596 SNPs. GWAS summary statistics for osteomyelitis were obtained from the FinnGen database (https://www.finngen.fi/en/access_results), which includes 1881 patients and 391,037 control individuals. In order to reduce the research bias caused by different ethnic groups, the data in the present study were drawn from individuals of European ancestry.

### IVs selection

The selection of Instrumental Variables (IVs) should satisfy three assumptions: First, instrumental variables must be closely related to exposure; Second, they are not associated with confounding factors, and then they can only influence outcomes through exposure. Therefore, the authors screened the IVs criteria for exposure phenotypes as follows: first, all SNPs contained in the IVs must be strongly related to exposure (p < 5e-08). Second, in order to reduce the influence of linkage disequilibrium, the SNPs with physical distance <10,000 kb or Linkage Disequilibrium (LD) potential R^2^ > 0.001 were removed by using the European population as a reference. Subsequently, the SNPs were also removed that were strongly related to the outcome (p < 5e-05). At the same time, the remaining SNPS were intersected with the SNPS in the resulting GWAS data to exclude the missing SNPS. To avoid the interference of reverse causality, the authors also eliminated the SNPs that failed the Steiger-filtering test and deleted the palindromes. Finally, the authors calculated the *F*-statistics of each SNP separately, and any SNPs with F-statistics <10 were excluded to ensure the strength of the IVs.

### Two-sample mendelian randomization

The “twoSampleMR” package (version 0.5.6) in the R program (version 4.2.3) was used for 2-SMR analysis. Three different methods of Inverse Variance Weighting (IVW), MR-Egger, and Weighted Median (WM) were used to evaluate the causal relationship of MR. The IVW method, as the most popular MR Method, is sensitive to potential impurities or trends between different genetic variants. The MR-Egger method can still provide consistent estimates when there is potential pleiotropy, but its statistical efficiency is insufficient, and it is susceptible to outlier SNPS^1^ The WM method is a relatively robust method that can provide consistent estimation as long as 50% of the SNPs are effective. For each method, a two-tailed p-value < 0.05 was considered indicative of statistical significance.

### Reverse MR

Reverse MR was used to evaluate the possibility of reverse causality of hyperthyroidism and hypothyroidism with osteomyelitis. To put it simply, reverse MR is an MR analysis that takes osteomyelitis as exposure and ends in hyperthyroidism and hypothyroidism Its screening criteria, implementation steps, and analysis methods for IVs are the same as those of 2-SMR.

### Sensitivity analysis

The authors used a variety of sensitivity analysis methods to assess the robustness of the results. The authors used Cochran's *Q*-test and funnel plot to detect whether there was heterogeneity among IVs SNPS, and a two-tailed p < 0.05 was considered heterogeneity. The authors use the intercept term obtained by MR-Egger regression to evaluate the pleiotropy. To be specific, when p < 0.05 and the Egger intercept is not 0, it may indicate the existence of horizontal pleiotropy; on the contrary, when the Egger intercept is close to 0 and the p-value is large, much larger than the usual significance level (0.05), it indicates that no significant horizontal pleiotropy can be found in this horizontal pleiotropy test. Finally, through visualization, the leave-one-out test was also used to evaluate whether there were outlier SNPs that significantly affected causal assessment.

### Multivariable MR

The authors conducted an MVMR analysis to assess whether hyperthyroidism and hypothyroidism is still the cause of osteomyelitis after adjusting for the effects of years of schooling, smoking initiation, and BMI. The specific method included the selection of years of schooling, smoking initiation, BMI and SNPs with a p-value < 5e-08 in the hypothyroidism phenotype. After removing duplicate SNPs, the authors eliminated the SNPs with a physical distance < 10,000 kb and LD likelihood R^2^ > 0.001. Then, the authors removed the palindrome SNPs and extracted the remaining SNPs from the resulting GWAS data, excluding any missing SNPs. Finally, the authors conducted an MVMR analysis in R.

## Results

### Two-way causal relationship between hyperthyroidism, hypothyroidism and osteomyelitis (UVMR analysis)

IVW method in MR Analysis was used to examine the two-way causal relationship between hyperthyroidism, hypothyroidism, and osteomyelitis. The results indicated that both of them exhibited a positive causal association with osteomyelitis, and there was no reverse causal relationship. In the forward MR analysis, 11 SNPs and 63 SNPs were selected as IVs of hyperthyroidism and hypothyroidism, respectively. All included SNPS can be viewed in Supplemental Tables 1 and 2.

**Fig. 1:**
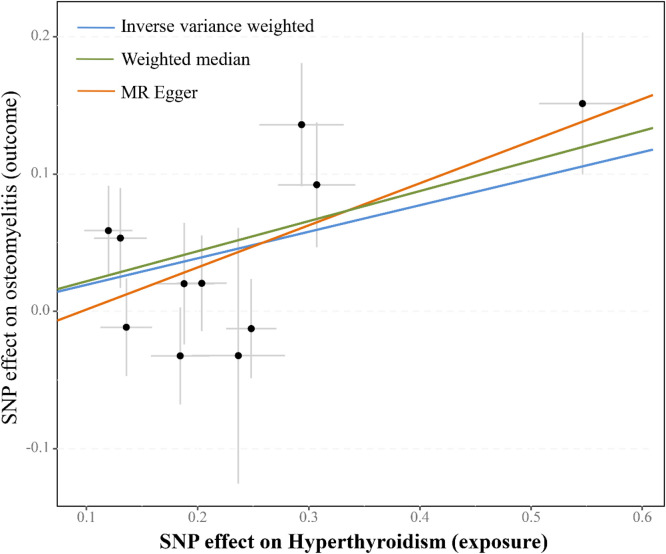
Scatter plots of univariable Mendelian randomization tests assessing the effect of hyperthyroidism on osteomyelitis. Each dot represents effect sizes of each Single Nucleotide Polymorphism (SNP) on hyperthyroidism (x‐axis) and osteomyelitis (y‐axis), and regression slopes show the pooled causal effect. For the plot, different MR methods (Inverse variance weighted, MR Egger and Weighted median) were visualized using different colors.

The results showed that the increase of Hyperthyroidism (OR = 1.21, 95% CI 1.071‒1.374, p = 0.002) was the cause of osteomyelitis, and was further supported by the WM method (OR = 1.25, 95% CI 1.073‒1.445; p = 0.004). The correlation direction obtained by the MR-Egger method is consistent with that obtained by the IVW and WM methods (OR = 1.36, 95% CI 1.013‒1.822; p = 0.07). The scatter plots displayed the slope of each fitted line representing the pooled causal relationship from each MR method (Fig. 1). The UVMR analysis results of hyperthyroidism and osteomyelitis are shown in Table 1. Sensitivity analysis ensures the stability of these results. The results of heterogeneity analysis (p > 0.05) and horizontal pleiotropy analysis (p > 0.05) indicate the stability of the final results. The leave-one-out test showed that there were no outliers in the IVs that significantly affected the results (Supplemental Fig. 1). The results of heterogeneity analysis, horizontal pleiotropy analysis, and leave-one-out test can be viewed in Supplemental File 1.

**Table 1:** The univariable Mendelian randomization analysis results of hyperthyroidism (exposure) and osteomyelitis (outcome).

Method	Number of SNP	Beta	OR (95% CI)	p
Inverse variance weighted	11	0.193	1.213 (1.072, 1.374)	0.002
MR Egger	11	0.307	1.359 (1.013, 1.822)	0.071
Weighted median	11	0.219	1.245(1.073, 1.445	0.004

OR, Odds Ratio; CI, Confidence Interval.

**Fig. 2:**
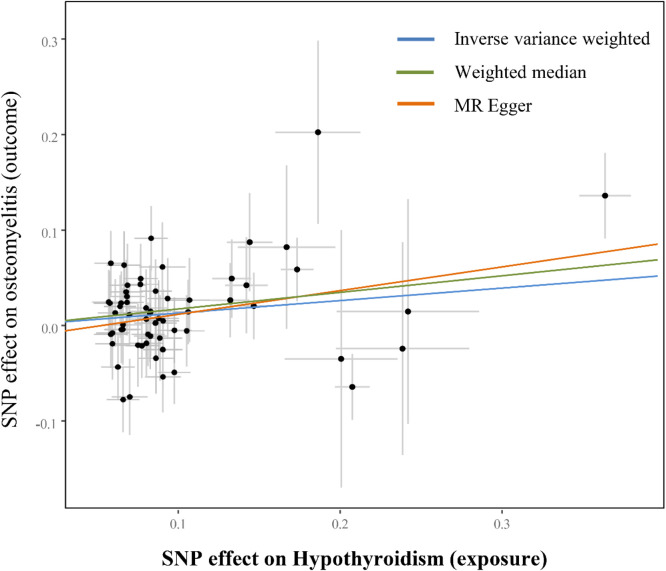
Scatter plots of univariable Mendelian randomization tests assessing the effect of hypothyroidism on osteomyelitis. Each dot represents effect sizes of each Single Nucleotide Polymorphism (SNP) on hypothyroidism (x‐axis) and osteomyelitis (y‐axis), and regression slopes show the pooled causal effect. For the plot, different MR methods (Inverse variance weighted, MR Egger, Weighted median and Weighted mode) were visualized using different colors.

The increase of hypothyroidism (OR = 1.140, 95% CI 1.035‒1.255, p = 0.0078) was the cause of osteomyelitis, and was further supported by the WM method (OR = 1.190, 95% CI 1.03‒1.38; p = 0.02). Meanwhile, the correlation direction obtained by the MR-Egger method is consistent with that obtained by the IVW and WM methods (OR = 1.28, 95% CI 1.03‒1.60; p = 0.03). The scatter plots displayed the slope of each fitted line representing the pooled causal relationship from each MR method (Fig. 2). The UVMR analysis results of hypothyroidism and osteomyelitis are shown in Table 2. The authors also performed a sensitivity analysis to ensure the stability of the present results. The results of heterogeneity analysis (p > 0.05) and horizontal pleiotropy analysis (p > 0.05) indicate the stability of the final results. The leave-one-out test showed that there were no outliers in the IVs that significantly affected the results (Supplemental Fig. 2). The results of heterogeneity analysis, horizontal pleiotropy analysis and leave-one-out test can be viewed in Supplemental File 2.

**Table 2:** The univariable Mendelian randomization analysis results of hypothyroidism (exposure) and osteomyelitis (outcome).

Method	Number of SNP	Beta	OR (95% CI)	p
Inverse variance weighted	63	0.131	1.140(1.035, 1.255)	0.008
MR Egger	63	0.248	1.281(1.027, 1.599)	0.032
Weighted median	63	0.174	1.190(1.026, 1.379)	0.021
Weighted mode	63	0.31	1.363(1.098, 1.692)	0.007

OR, Odds Ratio; CI, Confidence Interval.

### Causal relationship between hyperthyroidism, hypothyroidism and osteomyelitis (MVMR analysis)

**Fig. 3:**
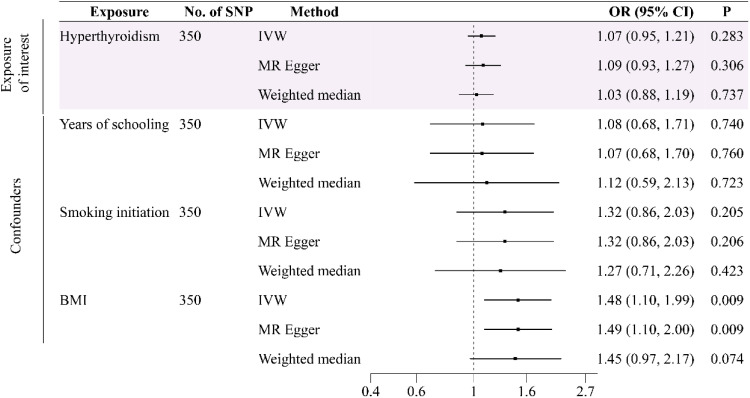
Forest plot of multivariable Mendelian randomization tests assessing the effect of hyperthyroidism on osteomyelitis with adjustment of years of schooling, smoking initiation and BMI.

**Fig. 4:**
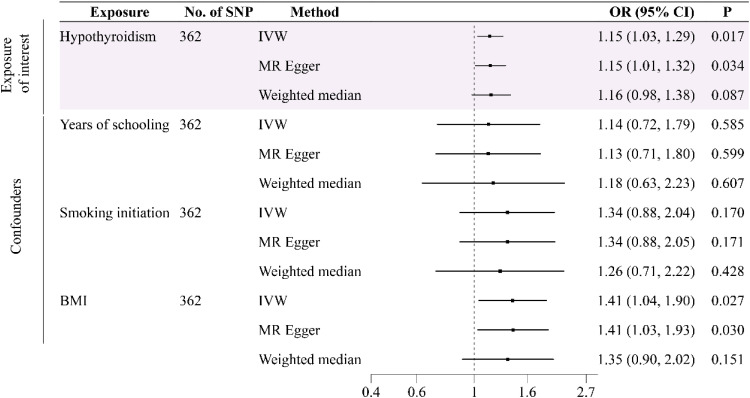
Forest plot of multivariable Mendelian randomization tests assessing the effect of hypothyroidism on osteomyelitis with adjustment of years of schooling, smoking initiation and BMI.

In order to reduce the influence of other factors on the causal relationship between hyperthyroidism, hypothyroidism, and osteomyelitis, the authors also performed a multivariate Mendelian randomization. A total of 350 and 362 SNPs were selected as IVs to be included in the MVMR analysis, respectively. The results showed that there was no causal relationship between hyperthyroidism (350 SNPs) and osteomyelitis (OR = 1.07, 95% CI 0.95‒1.21, p = 0.28) after removing confounding factors (Fig. 3), but there was still a strong risk factor affecting osteomyelitis (OR = 1.15, 95% CI 1.03‒1.29, p = 0.02) after adjusting for the effects of years of schooling, smoking initiation and BMI (362 SNPs for hypothyroidism). The results of the MVMR analysis were presented in Fig. 4. Although heterogeneity analysis (p < 0.05) showed that heterogeneity may exist, it did not affect the results. The authors used a random effects model to reduce the impact on the results. No horizontal pleiotropy was found in the sensitivity analysis (p > 0.05).

## Discussion

In this study, the authors integrated multiple MR methods harnessing genetic variability to investigate the causal associations between thyroid dysfunction and osteomyelitis. The present data in two-sample MR demonstrated that hypothyroidism and hyperthyroidism are both significantly associated with an increased risk of osteomyelitis. However, the independent causal effect between hypothyroidism and osteomyelitis was observed only after adjustment for years of schooling, smoking initiation and BMI jointly, suggesting that hypothyroidism could be an independent risk factor for the onset of osteomyelitis. In contrast, the reverse MR analysis did not show any indication that liability to hyperthyroidism or hypothyroidism was related to osteomyelitis. These findings on the causal relationship between thyroid dysfunction (especially hypothyroidism) and osteomyelitis offer fresh perspectives on the etiology of osteomyelitis.

Thyroid hormones are essential for the regulation of cellular metabolism, growth, and differentiation. Hypothyroidism, a prevalent endocrine disorder, arises from diminished synthesis and secretion of thyroid hormone or inadequate physiological response to thyroid hormone, manifesting primarily as subclinical hypothyroidism and overt hypothyroidism. Epidemiological data indicate a prevalence of 4.70% in Europe and 13.95% in the general population of China^12^^,^^13^ Endocrine-immune interaction makes the bidirectional communication between the endocrine and immune systems. A study found that a higher prevalence of thyroid dysfunction, particularly hypothyroidism, in patients with autoimmune disorders.^14^ In recent years, there has been extensive publicity regarding the significant correlation between hypothyroidism and infectious disease. In a 2014 study by Jung et al. in Korea, a survey was conducted on 235 patients undergoing peritoneal dialysis, which indicated that lower fT4 levels were predictive of mortality due to infection.^15^ Subsequently, Nobrega et al. conducted a study on Bacteriuria and urinary tract infection following a female urodynamic study, revealing an association between hypothyroidism and an increased risk of bacteriuria post-urodynamic examination.^16^ In 2016, a study was conducted on a sample of 32,289 participants to examine the correlation between hypothyroidism and periprosthetic joint infections following total joint arthroplasty. The results indicated that individuals diagnosed with hypothyroidism had a higher odds ratio for periprosthetic joint infection, suggesting that hypothyroidism may be an independent risk factor.^17^ More recently, the Norwegian prospective population-based HUNT Study investigated the relationship between thyroid function and the risk of bloodstream infections, finding that hypothyroidism was associated with a 20% increased risk of severe bloodstream infections.^18^ The aforementioned studies indicate a strong association between hypothyroidism and infectious diseases, suggesting that hypothyroidism may serve as an independent risk factor for such conditions. Nevertheless, the absence of observational studies on the relationship between hypothyroidism and osteomyelitis precludes any definitive conclusions regarding this specific correlation.

In the present investigation, the authors have confirmed that hypothyroidism serves as a contributing factor to osteomyelitis through the utilization of univariable and multivariable MR approaches, incorporating the latest genetic data derived from GWAS. This aligns with a broader understanding that thyroid hormones are vital for the normal function of multiple organ systems, and their dysregulation can have significant repercussions, for instance, on the reproductive system, potentially leading to subfertility.^19^ This finding extends this principle of systemic impact to the specific context of bone infections. The primary explanation for the elevated risk of osteomyelitis associated with hypothyroidism lies in the crucial role of thyroid hormone in regulating immune system homeostasis.^20^, ^21^, ^22^ Thyroid hormones can be involved in the activation and modulation of adaptive immune responses and the modulation of the function of immune cells. Thyroid hormone primarily exerts its effects through nuclear thyroid receptors, which regulate transcription factors involved in inflammation, such as NF-κB and JAK/STAT pathways.^23^ Additionally, thyroid hormones promote pro-inflammatory responses in macrophages and regulate their growth and metabolic activity.^24^ Studies have also shown a positive correlation between thyroid hormone concentrations and markers of inflammation.^25^ Therefore, individuals with hypothyroidism may experience reduced immune cell numbers and impaired function, resulting in compromised defense against infections and increased susceptibility to bacterial or other pathogenic agents. This increased susceptibility may consequently raise the likelihood of developing osteomyelitis. The mechanisms of this immune dysregulation are complex, potentially involving not only direct signaling pathways but also the thyroid gland's high intrinsic level of oxidative stress, which is influenced by other endocrine interactions. However, it is noteworthy that this immune effect may be specific, as one study found no significant association between hypothyroidism and a panel of general inflammatory hematological parameters. This suggests the pro-infective state the authors observed may not be reflected as a systemic inflammatory signature in routine blood work, but rather through more targeted pathways.^26^^,^^27^ According to the principles of osteoimmunology, preserving bone mass is essential for maintaining immune function. Zhang et al.'s cohort study revealed that individuals with low Bone Mineral Density (BMD), especially those with osteoporosis, have an increased susceptibility to infections and sepsis compared to those with normal BMD, suggesting that BMD is a newly identified risk factor for these conditions.^28^ It is well-established that thyroid hormones play a significant role in the regulation of bone mineral accumulation. Previous study has shown that individuals with hypothyroidism experience accelerated bone loss,^29^^,^^30^ resulting in decreased bone mass and potentially compromising the ability of body to combat infections, thus elevating the susceptibility to osteomyelitis. This is particularly relevant as thyroid dysfunction is a significant concern for bone health, especially in postmenopausal women who are already at an increased risk for osteoporosis. The established association between hypothyroidism and lower bone mineral density reinforces the biological plausibility that compromised bone integrity could create a favorable environment for infection^31^ In addition, hypothyroidism is often associated with autoimmune disorders,^32^, ^33^, ^34^ which would, by itself, predispose patients to osteomyelitis. Moreover, hypothyroidism itself can arise from autoimmune etiologies, such as Hashimoto's thyroiditis ‒ a common autoimmune thyroid disorder characterized by lymphocytic infiltration of the thyroid gland, ultimately leading to hypothyroidism.^35^

Particular emphasis should be placed on specific infections that have the potential to induce thyroid dysfunction. Prior observational research has indicated a higher prevalence of thyroid dysfunction in individuals with COVID-19,^36^ with subsequent MR studies revealing a direct link between SARS-CoV-2 viral infection and an elevated risk of hypothyroidism.^37^^,^^38^ In backward MR analysis, the possibility of thyroid dysfunction due to osteomyelitis has already been ruled out. Furthermore, this study validated the endocrine-immune interaction by validating the causal link between hypothyroidism and osteomyelitis. Future studies can investigate the interplay between thyroid dysfunction and other immune-related conditions, such as autoimmune diseases (e.g., rheumatoid arthritis and systemic lupus erythematosus) or chronic infections. Additionally, the authors suggest examining the role of other endocrine organs, such as the adrenal and pituitary glands, in immune regulation and their potential links to infectious diseases.

This study exhibits several notable strengths. Primarily, it is the inaugural study to establish a direct correlation between hypothyroidism and osteomyelitis, underscoring the autonomous influence of hypothyroidism on osteomyelitis development. Moreover, to enhance the accuracy of the causal inferences, an extensive and meticulous analysis employing bidirectional and multivariate MR techniques was undertaken. Furthermore, a variety of sensitivity analyses were conducted to verify the robustness and consistency of the causal relationship. In addition, the inclusion of a large number of SNPs as Instrumental Variables (IVs) bolsters the reliability of the findings.

While this study has notable strengths, it is important to acknowledge its limitations. Firstly, the participant pool was restricted to individuals of European descent, which may limit the generalizability of the findings to other ethnic populations. Future research incorporating diverse populations is necessary to validate these results and enhance their applicability across different genetic and environmental contexts. Secondly, the lack of data stratification by age and sex, particularly in the context of hypothyroidism being more prevalent in females, is a notable shortcoming. Thirdly, even though the authors conducted the multivariable MR analysis with adjustments for years of schooling, smoking initiation, and BMI, other potential confounders, such as physical activity, diet, and socioeconomic status, may also influence the observed relationship. Future studies should incorporate a broader range of confounders to enhance the robustness of the findings. Finally, while the bidirectional MR approach leverages genetic variants as instrumental variables to minimize confounding and can provide robust evidence for causal relationships, its validity depends on the validity of key assumptions in MR (relevance, independence and exclusion restriction).^39^ Weak instruments or those with pleiotropic effects may introduce bias, despite these efforts to select valid instruments. Residual confounding could still occur if genetic variants influence the outcome through pathways unrelated to the exposure. Furthermore, MR assumes a linear relationship between exposures (e.g., hypothyroidism) and outcomes (e.g., osteomyelitis). If this relationship were found to be non-linear, it could also impact the validity of the present findings. Even though the authors have conducted extensive sensitivity analyses to address these risks, these results should be interpreted with caution. Future studies with more comprehensive data and advanced methodologies are needed to further validate the present findings.

In summary, the MR method was employed to examine the potential causal relationship between hypothyroidism and osteomyelitis from a genetic standpoint. The results of this study have significant implications for informing clinical decision-making. Therefore, this study suggests that, in conjunction with established osteomyelitis risk factors such as diabetes or immunosuppression, clinicians should consider monitoring patients with hypothyroidism more closely during surgery, trauma, or infection, while individual risk assessment remains paramount. Ultimately, these findings contribute to the growing body of evidence underscoring the importance of maintaining “thyroid health”, a concept that warrants continued awareness among both clinicians and the public to prevent a wide range of associated morbidities^40^

## Clinical trial number

Not applicable

## Ethics approval and consent to participate

Not applicable

## Consent for publication

Not applicable

## Authors’ contributions

Junming Huang and Shanhu Huang conceived and revised the study. Junming Huang performed the experiments. Mingchao Lin and Shanhu Huang analyzed the data. Junming Huang and Zhiyuan Zou wrote the paper. Zhipeng Wang, Song Zhou and Shanhu Huang revised the manuscript. Every author has participated in the completion of the ultimate draft and given their approval for the release of the final document.

## Declaration of competing interest

The authors declare no conflicts of interest.

## Appendix Supplementary materials

Image, application 1

Image, application 2
