# Supplementary material for: Genetic evidence suggests a causal relationship linking thyroid function to osteomyelitis
Source: Clinics (Sao Paulo). 2026 Apr 23;81:100940. doi: 10.1016/j.clinsp.2026.100940 (PMC13126334; doi:10.1016/j.clinsp.2026.100940)
Supplement: Supplementary file 1 [file mmc1.docx]

**STROBE-MR checklist of recommended items to address in reports of Mendelian randomization studies**^1^ ^2^

| **Item No.** | **Section** | **Checklist item** | **Page No.** | **Relevant text from manuscript** |
| --- | --- | --- | --- | --- |
| 1 | **TITLE and ABSTRACT** | Indicate Mendelian randomization (MR) as the study’s design in the title and/or the abstract if that is a main purpose of the study | Page 2, line 41-46 | two-sample Mendelian randomization (MR), multivariable MR (MVMR), Supplementary backward MR analysis |
|  | **INTRODUCTION** |  |  |  |
| 2 | **Background** | Explain the scientific background and rationale for the reported study. What is the exposure? Is a potential causal relationship between exposure and outcome plausible? Justify why MR is a helpful method to address the study question | Page 3-4, line 87-106 | The onset of osteomyelitis is closely tied to external infectious factors. There is a difference in the bacterial profile of the infection depending on the age of the susceptible individual, including infants, children, and adults…Thus, we cannot make a conclusion about the causal relationship between thyroid dysfunction and osteomyelitis. |
| 3 | **Objectives** | State specific objectives clearly, including pre-specified causal hypotheses (if any). State that MR is a method that, under specific assumptions, intends to estimate causal effects | Page 4, line 115-119 | In this study, we wondered to acquire a thorough understanding of the causal link between thyroid dysfunction and osteomyelitis by conducting a two-sample MR analysis to explore the potential causal connection. The primary goal of our study is to offer stronger evidences and deeper insights into the pathophysiology of osteomyelitis, contributing to establish a scientific foundation for forthcoming prevention and treatment strategies. |
|  | **METHODS** |  |  |  |
| 4 | **Study design and data sources** | Present key elements of the study design early in the article. Consider including a table listing sources of data for all phases of the study. For each data source contributing to the analysis, describe the following: |  |  |
|  | a) | Setting: Describe the study design and the underlying population, if possible. Describe the setting, locations, and relevant dates, including periods of recruitment, exposure, follow-up, and data collection, when available. | Page 4, line 123-127 | Please check the “study design” |
|  | b) | Participants: Give the eligibility criteria, and the sources and methods of selection of participants. Report the sample size, and whether any power or sample size calculations were carried out prior to the main analysis | Page 4-5, line 130-141 | Please check the “GWAS data for hyperthyroidism, hypothyroidism, years of schooling, smoking initiation, BMI and osteomyelitis” |
|  | c) | Describe measurement, quality control and selection of genetic variants | Page 5, line 143-154 | Please check the “IVs selection” |
|  | d) | For each exposure, outcome, and other relevant variables, describe methods of assessment and diagnostic criteria for diseases | Page 4-5, line 130-141 | Please check the “GWAS data for hyperthyroidism, hypothyroidism, years of schooling, smoking initiation, BMI and osteomyelitis” |
|  | e) | Provide details of ethics committee approval and participant informed consent, if relevant | NA |  |
| 5 | **Assumptions** | Explicitly state the three core IV assumptions for the main analysis (relevance, independence and exclusion restriction) as well assumptions for any additional or sensitivity analysis | Page 5, line 143-154 | Please check the “IVs selection” |
| 6 | **Statistical methods: main analysis** | Describe statistical methods and statistics used |  |  |
|  | a) | Describe how quantitative variables were handled in the analyses (i.e., scale, units, model) | Page 5, line 156-168  Page 6, line 179-186 | Please check the “Two-sample Mendelian randomization”  Please check the “Reverse MR”  Please check the “Multivariable MR” |
|  | b) | Describe how genetic variants were handled in the analyses and, if applicable, how their weights were selected | Page 5, line 156-163 | The “two Sample MR” package (version 0.5.6) in the R program (version 4.2.3) was used for 2-SMR analysis. Three different methods of inverse variance weighting (IVW), MR-Egger and weighted median (WM) were used to evaluate the causal relationship of MR. The IVW method, as the most popular MR Method, is sensitive to potential impurities or trends between different genetic variants. The MR-Egger method can still provide consistent estimates when there is potential pleiotropy, but its statistical efficiency is insufficient and it is susceptible to outlier SNPS. The WM method is a relatively robust method that can provide consistent estimation as long as 50% of the SNPs are effective. For each method, a two-tailed P value < 0.05 was considered indicative of statistical significance. |
|  | c) | Describe the MR estimator (e.g. two-stage least squares, Wald ratio) and related statistics. Detail the included covariates and, in case of two-sample MR, whether the same covariate set was used for adjustment in the two samples | Page 5, line 156-163  Page 6, line 179-186 | Please check the “Two-sample Mendelian randomization”  Please check the “Multivariable MR” |
|  | d) | Explain how missing data were addressed | Page 5, line 150-154 | At the same time, the remaining SNPS were intersected with the SNPS in the resulting GWAS data to exclude the missing SNPS. To avoid the interference of reverse causality, we also eliminated the SNPs that failed the Steiger-filtering test and deleted the palindromes. Finally, we calculated the F statistics of each SNP separately, and any SNPs with F statistics less than 10 were excluded to ensure the strength of the IVs. |
|  | e) | If applicable, indicate how multiple testing was addressed | NA |  |
| 7 | **Assessment of assumptions** | Describe any methods or prior knowledge used to assess the assumptions or justify their validity | Page 3, line 102-104 | Previous studies have also demonstrated a strong association between thyroid dysfunction and susceptibility to infectious diseases, including pulmonary and urinary tract infections |
| 8 | **Sensitivity analyses and additional analyses** | Describe any sensitivity analyses or additional analyses performed (e.g. comparison of effect estimates from different approaches, independent replication, bias analytic techniques, validation of instruments, simulations) | Page 5-6, line 170-178 | Please check the “Sensitivity analysis” |
| 9 | **Software and pre-registration** |  |  |  |
|  | a) | Name statistical software and package(s), including version and settings used | Page 5, line 156-157 | The “two Sample MR” package (version 0.5.6) in the R program (version 4.2.3) was used for 2-SMR analysis. |
|  | b) | State whether the study protocol and details were pre-registered (as well as when and where) | NA |  |
|  | **RESULTS** |  |  |  |
| 10 | **Descriptive data** |  |  |  |
|  | a) | Report the numbers of individuals at each stage of included studies and reasons for exclusion. Consider use of a flow diagram | Page 4-5, line 130-141 | Please check the “GWAS data for hyperthyroidism, hypothyroidism, years of schooling, smoking initiation, BMI and osteomyelitis” |
|  | b) | Report summary statistics for phenotypic exposure(s), outcome(s), and other relevant variables (e.g. means, SDs, proportions) | NA |  |
|  | c) | If the data sources include meta-analyses of previous studies, provide the assessments of heterogeneity across these studies | NA |  |
|  | d) | For two-sample MR:  i.  Provide justification of the similarity of the genetic variant-exposure associations between the exposure and outcome samples  ii.  Provide information on the number of individuals who overlap between the exposure and outcome studies | Page 4-5, line 130-154 | Please check the “GWAS data for hyperthyroidism, hypothyroidism, years of schooling, smoking initiation, BMI and osteomyelitis”  Please check the “IVs selection” |
| 11 | **Main results** |  |  |  |
|  | a) | Report the associations between genetic variant and exposure, and between genetic variant and outcome, preferably on an interpretable scale | Page 6, line 191-195  Page 7, line 218-219 | IVW method in MR Analysis was used to examine the two-way causal relationship between hyperthyroidism, hypothyroidism and osteomyelitis. The results indicated that both of them exhibited a positive causal association with osteomyelitis, and there was no reverse causal relationship. In the forward MR analysis, 11 SNPs and 63 SNPs were selected as IVs of hypothyroidism and hypothyroidism respectively. All included SNPS can be viewed in Supplemental Table 1.  A total of 350 and 362 SNPs were selected as IVs to be included in the MVMR analysis, respectively. |
|  | b) | Report MR estimates of the relationship between exposure and outcome, and the measures of uncertainty from the MR analysis, on an interpretable scale, such as odds ratio or relative risk per SD difference | Page 6, line 196-198  Page 7, line 206-207 | The results showed that the increase of Hyperthyroidism (OR = 1.21, 95% CI 1.071-1.374, P = 0.002) was the cause of osteomyelitis, and was further supported by WM method (OR: 1.25, 95% CI 1.073, 1.445; P = 0.004).  The increase of hypothyroidism (OR = 1.140, 95% CI 1.035-1.255, P = 0.0078) was the cause of osteomyelitis, and was further supported by WM method (OR: 1.190, 95%CI 1.03, 1.38; P = 0.02). |
|  | c) | If relevant, consider translating estimates of relative risk into absolute risk for a meaningful time period | NA |  |
|  | d) | Consider plots to visualize results (e.g. forest plot, scatterplot of associations between genetic variants and outcome versus between genetic variants and exposure) | Page 6, line 199-200  Page 7, line 209-210 | The scatter plots displayed the slope of each fitted line representing the pooled causal relationship from each MR method (Figure 1)  The scatter plots displayed the slope of each fitted line representing the pooled causal relationship from each MR method (Figure 2) |
| 12 | **Assessment of assumptions** |  |  |  |
|  | a) | Report the assessment of the validity of the assumptions | Page 6, line 200-204  Page 7, line 211-213 | The UVMR analysis results of hyperthyroidism and osteomyelitis are shown in Table 1. sensitivity analysis to ensure the stability of our results. The results of heterogeneity analysis(P>0.05) and horizontal pleiotropy analysis(P>0.05) indicate the stability of our final results. The leave-one-out test showed that there were no outliers in the IVs that significantly affected the results.  We also performed sensitivity analysis to ensure the stability of our results. The results of heterogeneity analysis(P>0.05) and horizontal pleiotropy analysis(P>0.05) indicate the stability of our final results. |
|  | b) | Report any additional statistics (e.g., assessments of heterogeneity across genetic variants, such as *I^2^*, Q statistic or E-value) | Page 7, line 204-205, 213-215 | The results of heterogeneity analysis, horizontal pleiotropy analysis and leave-one-out test can be viewed in Supplemental Table 2.  The leave-one-out test showed that there were no outliers in the IVs that significantly affected the results. The results of heterogeneity analysis, horizontal pleiotropy analysis and leave-one-out test can be viewed in Supplemental Table 3. |
| 13 | **Sensitivity analyses and additional analyses** |  |  |  |
|  | a) | Report any sensitivity analyses to assess the robustness of the main results to violations of the assumptions | Page 6-7, line 196-215, 217-226 | Please check the “Two-way causal relationship between hyperthyroidism, hypothyroidism and osteomyelitis (UVMR analysis)”  Please check the “Causal relationship between hyperthyroidism, hypothyroidism and osteomyelitis (MVMR analysis)” |
|  | b) | Report results from other sensitivity analyses or additional analyses | Page 6-7, line 196-215, 217-226 | Please check the “Two-way causal relationship between hyperthyroidism, hypothyroidism and osteomyelitis (UVMR analysis)”  Please check the “Causal relationship between hyperthyroidism, hypothyroidism and osteomyelitis (MVMR analysis)” |
|  | c) | Report any assessment of direction of causal relationship (e.g., bidirectional MR) | Page 6-7, line 196-215, 217-226 | Please check the “Two-way causal relationship between hyperthyroidism, hypothyroidism and osteomyelitis (UVMR analysis)”  Please check the “Causal relationship between hyperthyroidism, hypothyroidism and osteomyelitis (MVMR analysis)” |
|  | d) | When relevant, report and compare with estimates from non-MR analyses | NA |  |
|  | e) | Consider additional plots to visualize results (e.g., leave-one-out analyses) | Page 6-7, line 196-215, 217-226 | Please check the “Two-way causal relationship between hyperthyroidism, hypothyroidism and osteomyelitis (UVMR analysis)”  Please check the “Causal relationship between hyperthyroidism, hypothyroidism and osteomyelitis (MVMR analysis)” |
|  | **DISCUSSION** |  |  |  |
| 14 | **Key results** | Summarize key results with reference to study objectives | Page 7, line 229-237 | In this study, we integrated multiple MR method harnessing genetic variability to investigate the causal associations between thyroid dysfunction and osteomyelitis. Our data in two-sample MR demonstrated that hypothyroidism and hyperthyroidism are both significantly associated with an increased risk of osteomyelitis. However, the direct causal correlation between hypothyroidism and osteomyelitis was only proposed when adjusted by years of schooling, smoking initiation and BMI jointly, suggesting that hypothyroidism could be an independent risk factor for the onset of osteomyelitis. In contrast, the reverse MR analysis did not show any indication that liability to hyperthyroidism or hypothyroidism was related to osteomyelitis. These findings on causal relationship between thyroid dysfunction (especially hypothyroidism) and osteomyelitis offer fresh perspectives on etiology of osteomyelitis. |
| 15 | **Limitations** | Discuss limitations of the study, taking into account the validity of the IV assumptions, other sources of potential bias, and imprecision. Discuss both direction and magnitude of any potential bias and any efforts to address them | Page 9, line 289-295 | While this study has notable strengths, it is important to acknowledge its limitations. Firstly, the participant pool was limited to individuals of European descent, which may hinder the applicability of the results to diverse ethnic populations. Moreover, the lack of data stratification by age and sex, particularly in the context of hypothyroidism being more prevalent in females, is a notable shortcoming. Finally, Mendelian randomization is based on the assumption of a linear relationship between exposure factors, such as hypothyroidism, and disease outcomes, such as osteomyelitis. If this relationship is found to be non-linear, it may impact the validity of using Mendelian randomization in the study. |
| 16 | **Interpretation** |  |  |  |
|  | a) | Meaning: Give a cautious overall interpretation of results in the context of their limitations and in comparison with other studies | Page 9, line 289-291 | Firstly, the participant pool was limited to individuals of European descent, which may hinder the applicability of the results to diverse ethnic populations. |
|  | b) | Mechanism: Discuss underlying biological mechanisms that could drive a potential causal relationship between the investigated exposure and the outcome, and whether the gene-environment equivalence assumption is reasonable. Use causal language carefully, clarifying that IV estimates may provide causal effects only under certain assumptions | Page 8-9, line 260-376 | Various potential explanations may account for the elevated risk of osteomyelitis associated with hypothyroidism. immune function…In addition, hypothyroidism is often associated with an autoimmune spectrum, which would, by itself, predispose patients to osteomyelitis. |
|  | c) | Clinical relevance: Discuss whether the results have clinical or public policy relevance, and to what extent they inform effect sizes of possible interventions | Page 9, line 297-300 | The results of this study have significant implications for informing clinical decision-making. Therefore, this study suggests that it is imperative to closely monitor the progression of patients with hypothyroidism undergoing surgery, trauma, and infectious illnesses, and to intervene effectively in a timely manner to prevent the onset of osteomyelitis. |
| 17 | **Generalizability** | Discuss the generalizability of the study results (a) to other populations, (b) across other exposure periods/timings, and (c) across other levels of exposure | Page 9, line 289-295 | Firstly, the participant pool was limited to individuals of European descent, which may hinder the applicability of the results to diverse ethnic populations. Moreover, the lack of data stratification by age and sex, particularly in the context of hypothyroidism being more prevalent in females, is a notable shortcoming. Finally, Mendelian randomization is based on the assumption of a linear relationship between exposure factors, such as hypothyroidism, and disease outcomes, such as osteomyelitis. If this relationship is found to be non-linear, it may impact the validity of using Mendelian randomization in the study. |
|  | **OTHER INFORMATION** |  |  |  |
| 18 | **Funding** | Describe sources of funding and the role of funders in the present study and, if applicable, sources of funding for the databases and original study or studies on which the present study is based | Page 10, line 319-327 | Please check the “Funding” |
| 19 | **Data and data sharing** | Provide the data used to perform all analyses or report where and how the data can be accessed, and reference these sources in the article. Provide the statistical code needed to reproduce the results in the article, or report whether the code is publicly accessible and if so, where | NA |  |
| 20 | **Conflicts of Interest** | All authors should declare all potential conflicts of interest | Page 1, line 26 | All authors declare no competing interests associated with this study. |

This checklist is copyrighted by the Equator Network under the Creative Commons Attribution 3.0 Unported (CC BY 3.0) license.

1. Skrivankova VW, Richmond RC, Woolf BAR, Yarmolinsky J, Davies NM, Swanson SA, et al. Strengthening the Reporting of Observational Studies in Epidemiology using Mendelian Randomization (STROBE-MR) Statement. JAMA. 2021;under review.

2. Skrivankova VW, Richmond RC, Woolf BAR, Davies NM, Swanson SA, VanderWeele TJ, et al. Strengthening the Reporting of Observational Studies in Epidemiology using Mendelian Randomisation (STROBE-MR): Explanation and Elaboration. BMJ. 2021;375:n2233.
