# Supplementary material for: Genetic evidence suggests a causal relationship linking thyroid function to osteomyelitis
Source: Clinics (Sao Paulo). 2026 Apr 23;81:100940. doi: 10.1016/j.clinsp.2026.100940 (PMC13126334; doi:10.1016/j.clinsp.2026.100940)

CLINICS-D-25-01065_Supplementary Material

**Supplemental Table 1** Included SNPs of hyperthyroidism in mendelian randomized analysis.

| **SNP** | **Effect allele (exposure)** | **Other allele (exposure)** | **Effect allele (outcome)** | **Other allele (outcome)** | **Beta. Exposure** | **Beta. Outcome** | **Eaf. Exposure** | **Eaf. Outcome** |
| --- | --- | --- | --- | --- | --- | --- | --- | --- |
| rs1794280 | T | A | T | A | 0.5463 | 0.151389 | 0.0985158 | 0.100469 |
| rs2160215 | C | T | C | T | 0.2483 | -0.0125711 | 0.425594 | 0.295535 |
| rs2856821 | C | T | C | T | -0.1879 | -0.0201996 | 0.196264 | 0.164459 |
| rs3087243 | A | G | A | G | -0.2038 | -0.0204793 | 0.380249 | 0.329847 |
| rs4338740 | C | T | C | T | 0.1844 | -0.0324174 | 0.207075 | 0.314269 |
| rs58722186 | T | C | T | C | 0.1359 | -0.0115606 | 0.302235 | 0.308979 |
| rs604912 | G | A | G | A | 0.1198 | 0.0588569 | 0.538777 | 0.512143 |
| rs6131010 | G | A | G | A | 0.1306 | 0.0534028 | 0.712807 | 0.716206 |
| rs6679677 | A | C | A | C | 0.2936 | 0.13603 | 0.111921 | 0.146453 |
| rs758778 | C | T | C | T | 0.3072 | 0.0922475 | 0.203646 | 0.152294 |
| rs9258222 | A | G | A | G | -0.2367 | 0.0322622 | 0.0868963 | 0.0321941 |

**Supplemental Table 2** Included SNPs of hypothyroidism in mendelian randomized analysis.

| **SNP** | **Effect allele (exposure)** | **Other allele (exposure)** | **Effect allele (outcome)** | **Other allele (outcome)** | **Beta. Exposure** | **Beta. Outcome** | **Eaf. Exposure** | **Eaf. Outcome** |
| --- | --- | --- | --- | --- | --- | --- | --- | --- |
| rs10075764 | G | A | G | A | -0.057 | -0.02463 | 0.302413 | 0.39524 |
| rs10126000 | A | C | A | C | -0.0683 | -0.0242472 | 0.688692 | 0.659996 |
| rs10424978 | A | C | A | C | -0.0775 | 0.0212629 | 0.624328 | 0.59874 |
| rs1079418 | G | A | G | A | -0.0657 | 0.00405553 | 0.262374 | 0.26237 |
| rs10917477 | G | A | G | A | 0.064 | 0.0200288 | 0.386901 | 0.504711 |
| rs11171710 | A | G | A | G | -0.0698 | -0.0113153 | 0.443916 | 0.448459 |
| rs114285740 | C | G | C | G | 0.1669 | 0.0821311 | 0.0273776 | 0.0388485 |
| rs11675342 | T | C | T | C | 0.0906 | 0.00491055 | 0.387729 | 0.440811 |
| rs11875260 | G | A | G | A | 0.0751 | -0.0205695 | 0.172925 | 0.172582 |
| rs12117927 | A | C | A | C | 0.0627 | -0.0434761 | 0.476541 | 0.405227 |
| rs12379417 | A | G | A | G | 0.0583 | 0.0653092 | 0.317559 | 0.377692 |
| rs12582330 | T | G | T | G | -0.061 | -0.0132438 | 0.624365 | 0.67596 |
| rs12593201 | A | G | A | G | 0.0905 | -0.0537429 | 0.325432 | 0.262458 |
| rs12984428 | A | G | A | G | -0.0659 | 0.0776207 | 0.35604 | 0.363686 |
| rs13090803 | T | G | T | G | 0.0829 | 0.0147996 | 0.190624 | 0.140013 |
| rs13109179 | A | G | A | G | 0.0647 | 0.0236041 | 0.461895 | 0.472439 |
| rs1364450 | C | A | C | A | 0.0886 | -0.0131594 | 0.126235 | 0.16023 |
| rs142997491 | G | A | G | A | 0.2385 | -0.0241898 | 0.0135637 | 0.0223971 |
| rs1432806 | G | A | G | A | 0.0583 | -0.00925879 | 0.338619 | 0.307265 |
| rs1479565 | A | G | A | G | 0.0975 | -0.0490716 | 0.498866 | 0.41819 |
| rs1534430 | T | C | T | C | -0.086 | -0.0360415 | 0.428394 | 0.418239 |
| rs187707293 | A | T | A | T | 0.2419 | 0.0146914 | 0.0133864 | 0.0211048 |
| rs2111485 | G | A | G | A | 0.0813 | -0.00919479 | 0.479023 | 0.58266 |
| rs2114702 | A | T | A | T | 0.07 | -0.0747311 | 0.26671 | 0.216086 |
| rs2234167 | A | G | A | G | 0.0825 | 0.0125943 | 0.102013 | 0.12081 |
| rs2247314 | C | T | C | T | -0.086 | -0.00274655 | 0.376495 | 0.370955 |
| rs229528 | T | C | T | C | 0.0903 | -0.0251189 | 0.496016 | 0.396236 |
| rs2445608 | A | G | A | G | -0.0593 | 0.00776396 | 0.430366 | 0.390037 |
| rs244685 | G | T | G | T | -0.0858 | -0.00252525 | 0.79915 | 0.747649 |
| rs2988277 | T | C | T | C | 0.0593 | -0.0191355 | 0.287022 | 0.258503 |
| rs3087243 | A | G | A | G | -0.1466 | -0.0204793 | 0.386088 | 0.329847 |
| rs3118469 | T | A | T | A | 0.0803 | -0.0185345 | 0.295174 | 0.363973 |
| rs3184504 | C | T | C | T | -0.1734 | -0.058703 | 0.668513 | 0.592574 |
| rs34536443 | C | G | C | G | -0.1863 | -0.202422 | 0.0440279 | 0.0301554 |
| rs3775291 | T | C | T | C | -0.0649 | 0.00421319 | 0.287788 | 0.316136 |
| rs434294 | G | A | G | A | -0.0683 | -0.0303832 | 0.322802 | 0.315703 |
| rs4409785 | C | T | C | T | 0.1069 | 0.0266321 | 0.142566 | 0.16624 |
| rs4529854 | T | C | T | C | -0.0768 | -0.049105 | 0.723438 | 0.720954 |
| rs4835534 | C | T | C | T | -0.1421 | -0.0422069 | 0.156356 | 0.123753 |
| rs61759532 | T | C | T | C | 0.0905 | -0.0252857 | 0.188941 | 0.189045 |
| rs61877856 | T | C | T | C | -0.0658 | -0.000712855 | 0.197495 | 0.228959 |
| rs6679677 | A | C | A | C | 0.3637 | 0.13603 | 0.108444 | 0.146453 |
| rs6908626 | T | G | T | G | 0.1441 | 0.087297 | 0.17075 | 0.115124 |
| rs7030280 | T | C | T | C | 0.2075 | -0.0642673 | 0.745664 | 0.653333 |
| rs71508903 | T | C | T | C | 0.0934 | 0.0283211 | 0.210816 | 0.185269 |
| rs7223956 | C | T | C | T | -0.0902 | -0.0613257 | 0.897005 | 0.855466 |
| rs73192661 | T | C | T | C | -0.1061 | -0.0142928 | 0.428858 | 0.385269 |
| rs736374 | A | G | A | G | 0.0832 | 0.0913816 | 0.375103 | 0.350099 |
| rs7441808 | G | A | G | A | 0.0766 | 0.043162 | 0.21091 | 0.282189 |
| rs7488011 | T | C | T | C | 0.1052 | -0.00558214 | 0.356485 | 0.256919 |
| rs7574865 | G | T | G | T | -0.1321 | -0.026534 | 0.74252 | 0.767846 |
| rs7742626 | C | T | C | T | 0.0686 | 0.0421959 | 0.267343 | 0.174372 |
| rs79490353 | C | T | C | T | 0.2006 | -0.0349061 | 0.0241151 | 0.0160156 |
| rs7990020 | C | A | C | A | 0.0577 | 0.0234064 | 0.453482 | 0.618923 |
| rs853305 | C | T | C | T | -0.0802 | -0.00677085 | 0.718894 | 0.788366 |
| rs881858 | A | G | A | G | 0.0665 | 0.0632866 | 0.747899 | 0.679005 |
| rs911760 | A | C | A | C | 0.0879 | 0.00759835 | 0.212143 | 0.225238 |
| rs926103 | C | T | C | T | -0.0678 | -0.0352244 | 0.697985 | 0.618744 |
| rs9264277 | C | T | C | T | -0.0862 | 0.0342807 | 0.645071 | 0.729561 |
| rs9277559 | C | T | C | T | -0.133 | -0.0492082 | 0.331026 | 0.197758 |
| rs9497965 | T | C | T | C | 0.0827 | -0.0109879 | 0.409035 | 0.336874 |
| rs9511151 | A | G | A | G | -0.0976 | 0.00494164 | 0.279567 | 0.344011 |
| rs9902341 | T | C | T | C | 0.0801 | 0.0183383 | 0.172257 | 0.203062 |

**Supplemental Figure 1** Leave-one-out sensitivity analysis results of hyperthyroidism (exposure) and osteomyelitis (outcome).


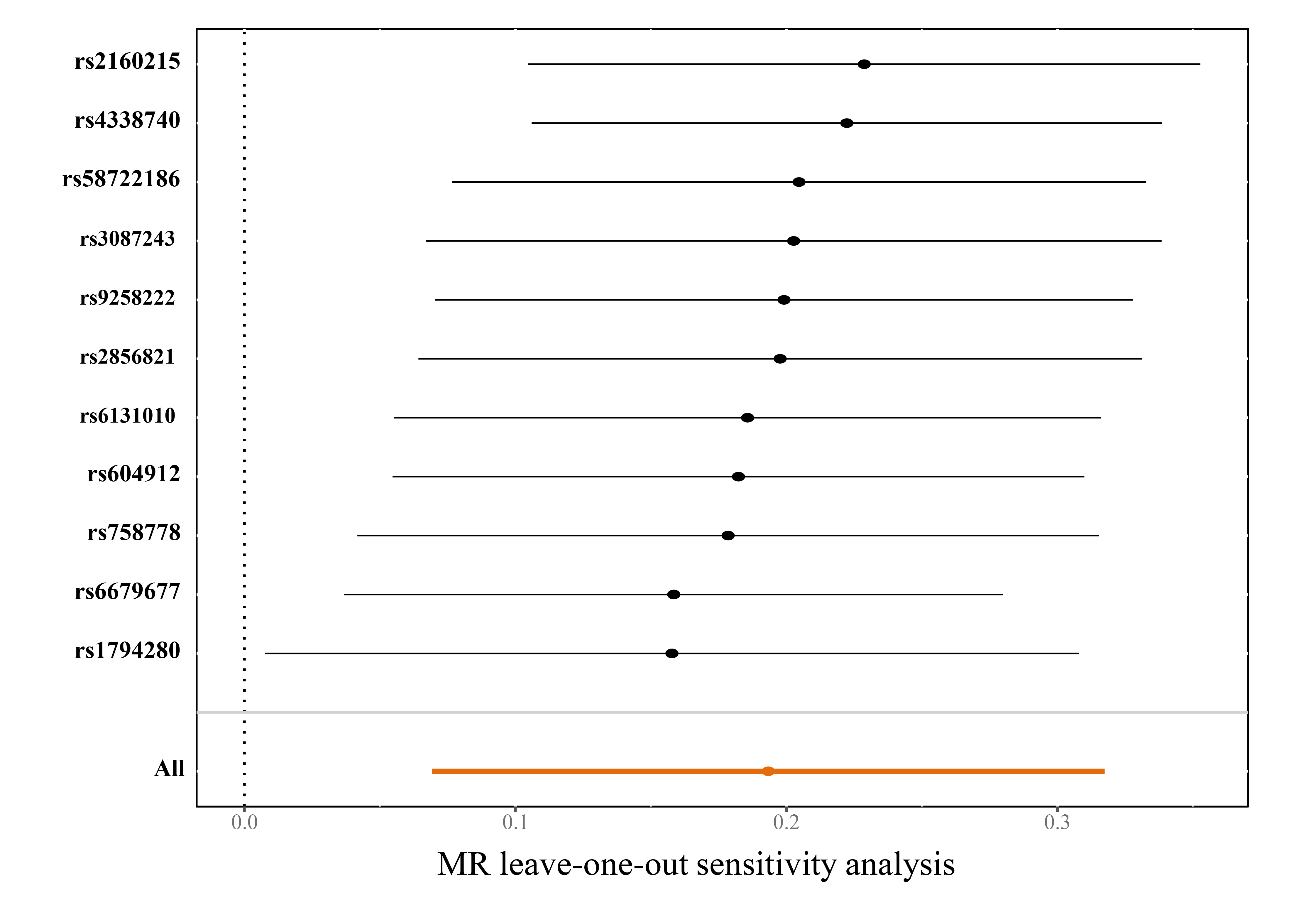


**Supplemental Figure 2** Leave-one-out sensitivity analysis results of hypothyroidism (exposure) and osteomyelitis (outcome).


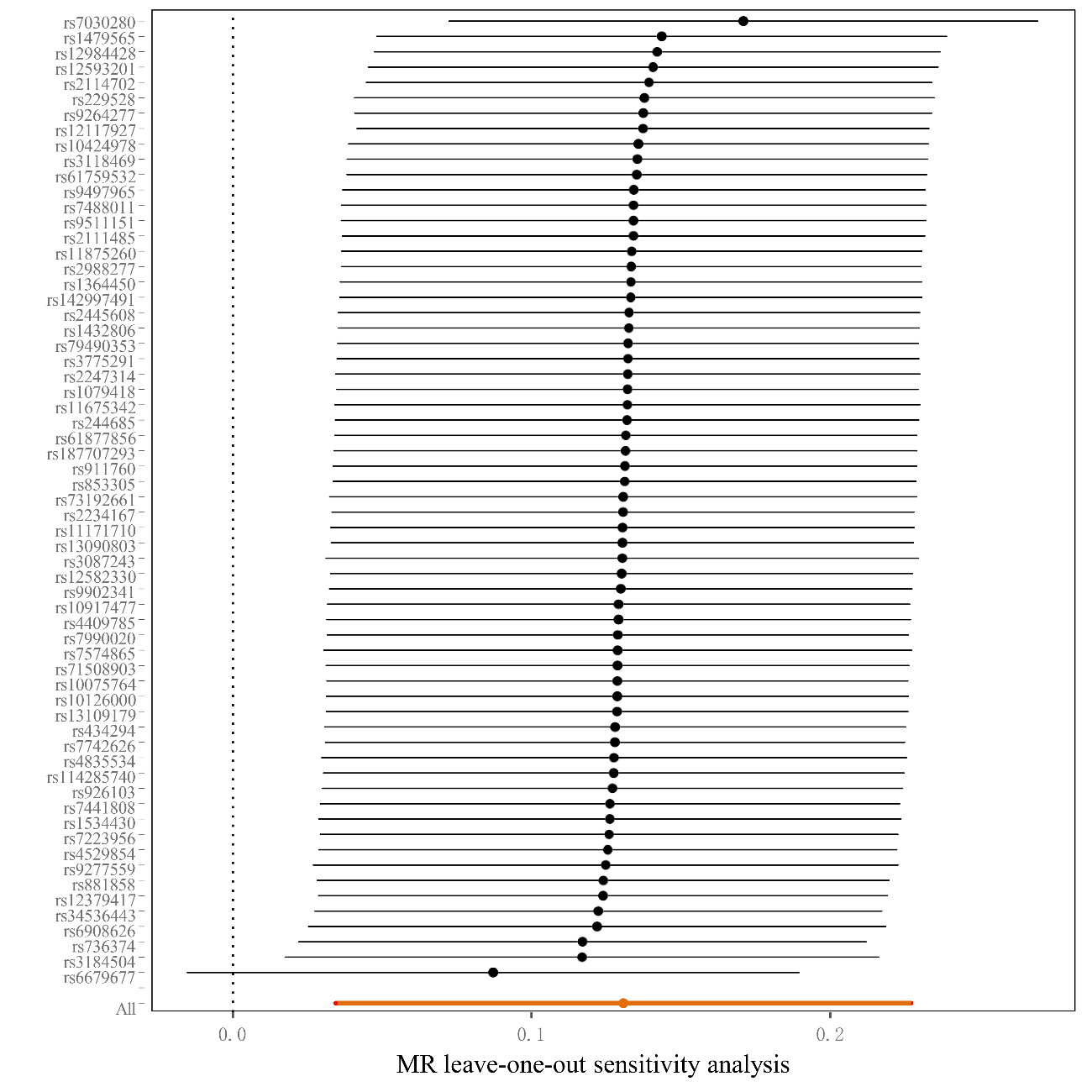

Supplement: Supplementary file 2 [file mmc2.docx]
